# Multicenter Study of Albuterol Use Among Infants Hospitalized with Bronchiolitis

**DOI:** 10.5811/westjem.2018.3.35837

**Published:** 2018-04-05

**Authors:** Anna Condella, Jonathan M. Mansbach, Kohei Hasegawa, Peter S. Dayan, Ashley F. Sullivan, Janice A. Espinola, Carlos A. Camargo

**Affiliations:** * Columbia University College of Physicians and Surgeons, Division of Pediatric Emergency Medicine, Department of Pediatrics, New York, New York; † Harvard Medical School, Boston Children’s Hospital, Department of Medicine, Boston, Massachusetts; ‡ Harvard Medical School, Massachusetts General Hospital, Department of Emergency Medicine, Boston, Massachusetts

## Abstract

**Introduction:**

Although bronchiolitis is a common reason for infant hospitalization, significant heterogeneity persists in its management. The American Academy of Pediatrics currently recommends that inhaled albuterol not be used in routine care of children with bronchiolitis. Our objective was to identify factors associated with pre-admission (e.g., emergency department or primary care) use of albuterol among infants hospitalized for bronchiolitis.

**Methods:**

We analyzed data from a 17-center observational study of 1,016 infants (age <1 year) hospitalized with bronchiolitis between 2011–2014. Pre-admission albuterol use was ascertained by chart review, and data were available for 1,008 (99%) infants. We used multivariable logistic regression to identify infant characteristics independently associated with pre-admission albuterol use.

**Results:**

Half of the infants (n=508) received at least one albuterol treatment before admission. Across the 17 hospitals, pre-admission albuterol use ranged from 23–84%. In adjusted analysis, independent predictors of albuterol use were the following: age ≥2 months (age 2.0–5.9 months [odds ratio (OR) 2.09, 95% confidence interval (CI) {1.45–3.01}] and age 6.0–11.9 months [OR 2.89, 95% CI {1.99–4.19}]); prior use of a bronchodilator (OR 1.89, 95% CI [1.24–2.90]); and presence of wheezing documented in pre-admission chart (OR 3.94, 95% CI [2.61–5.93]). By contrast, albuterol use was less likely among those with ≥7 days since the start of breathing problem (OR 0.66, 95% CI [0.44–1.00]) and parent-reported fever (OR 0.75, 95% CI [0.58–0.96]).

**Conclusion:**

Variation in pre-admission albuterol use suggests that local practice had a strong influence on use, but that patient characteristics also influenced the decision. While we agree with current guidelines in recommending against albuterol for all infants with bronchiolitis, our understanding of possible subgroups of responders may improve through investigation of infants with the identified characteristics.

## INTRODUCTION

Bronchiolitis is the most common cause for hospitalization of infants in the United States, with over 100,000 hospitalizations annually, representing approximately 3% of all children during their first year of life.^1^,^2^ In recent years, the number of annual visits to the emergency department (ED) for bronchiolitis has been increasing.^3^ Infants with bronchiolitis also have been found to have an increased likelihood of developing asthma.^4^–^8^ Bronchiolitis, therefore, affects a significant proportion of the population and is linked to further development of disease in that population. Nevertheless, clinical management of bronchiolitis is still highly variable.^9^–^11^ Although the variation of treatment for bronchiolitis is well-established and is a driving factor behind current clinical guidelines, little is known about how patient characteristics are associated with this variation.

The American Academy of Pediatrics (AAP) has published clinical guidelines, most recently updated in 2014, which recommend against the routine use of all pre-admission medications.^12^ Recent analyses on practice variation in the management of bronchiolitis have also taken a broad approach, establishing that variation occurs in the use of several therapies and diagnostic tests across different hospitals.^9^,^13^,^14^ In the current analysis, we have focused on a single therapy, inhaled albuterol, and the factors associated with its use. We conducted a secondary analysis of data from a prospective multicenter, multiyear study of over 1,000 infants. Our aim was to assess the variation across the 17 participating hospitals and identify patient characteristics independently associated with pre-admission albuterol use. We hypothesized that albuterol use would be common, with significant local variation, and associated with patient characteristics that suggest chronic breathing problems (e.g., older infant age, previous respiratory hospitalization, family history of asthma).

## METHODS

### Study Design

As part of the Multicenter Airway Research Collaboration, a clinical research program focusing on respiratory/allergy emergencies, the Emergency Medicine Network (www.emnet-usa.org) is conducting a multicenter, prospective cohort study that enrolled infants for three consecutive fall/winter seasons from 2011–2014. The total number of hospitals participating is 17, spread across 14 U.S. states. Evaluation and treatment of patients was at the discretion of the healthcare providers on site. Investigators enrolled patients using a standardized protocol. Inclusion criteria for the study were the following: an attending physician’s diagnosis of bronchiolitis (as defined by the AAP: an acute respiratory illness with some combination of rhinitis, wheezing, cough, tachypnea, crackles and retractions^15^); age of <1 year; a parent/guardian with the ability to give informed consent who spoke English or Spanish within 24 hours of admission; and complete contact information that was not expected to change for at least 12 months. Exclusion criteria included transfer to a participating hospital >48 hours after original admission, >24 hours since transferring to a participating hospital, a parent/guardian refusing collection or future use of biospecimens, insurmountable language barrier, certain chronic conditions (e.g., known heart-lung disease, immunodeficiency), gestational age <32 weeks, or the patient had met the primary endpoint of the initial five-year grant (U01 AI-087881) at the time of enrollment (i.e., two or more treatments with corticosteroids in six months, or four or more episodes of wheezing in one year). All participating hospitals had approval of their local institutional review board.

**Population Health Research Capsule**

What do we already know about this issue?

Bronchiolitis is a major cause of infant hospitalization but heterogeneity persists in its management. Current guidelines recommend against routine use of albuterol.

What was the research question?

What patient characteristics are associated with clinician use of pre-admission albuterol?

What was the major finding of the study?

Older age, prior use of a bronchodilator, and documented wheezing were associated with receiving pre-admission albuterol.

How does this improve population health?

Our understanding of variation in albuterol use and possible subgroups of responders may improve through investigation of infants with the identified characteristics.

### Data Collection

Investigators completed a structured interview with parents/guardians to assess patients’ demographic characteristics and history, and to obtain detailed information regarding the bronchiolitis episode for which they were admitted. Further clinical data on the patient’s evaluation, treatment and course of illness was obtained via the patient’s medical records. These data were abstracted from the medical record and entered into a standardized form by staff at EMNet. This chart review included the primary outcome of the current analysis, inhaled albuterol at any point during the entire pre-admission visit (e.g., in the ED of the enrolling hospital, the ED of another hospital, the primary care provider’s office, given during transfer, or another location such as an outpatient clinic or urgent care). With this variable, we specifically sought to identify whether or not a clinician chose to administer albuterol. Staff at EMNet Coordinating Center manually reviewed all data for any inconsistencies or missing information and then queried hospitals for clarification.

### Nasopharyngeal Aspirate and Virology

Nasopharyngeal aspirates were collected following a standardized procedure within 24 hours of admission for each participant.^16^ All samples were placed on ice and stored at −80°C. Polymerase chain reaction assays were performed as either singleplex or duplex two-step, real-time polymerase chain reactions. Aspirates were tested for a panel of common respiratory viruses, including respiratory syncytial virus (RSV) types A and B and rhinovirus (RV). The virology protocol is described elsewhere.^16^

### Statistical Analyses

We performed all analyses using Stata 14.1 (Stata Corp, College Station, TX). Data are presented as proportions with 95% confidence intervals (CI) and medians with interquartile ranges (IQR). To examine factors potentially associated with the primary outcome – pre-admission albuterol use among infants hospitalized for bronchiolitis – we performed unadjusted analyses using chi square, Fisher’s exact test, or Wilcoxon rank-sum test, as appropriate. All *P*-values were two-tailed, with *P*<0.05 considered statistically significant.

We conducted multivariable logistic regression to evaluate independent predictors of pre-admission albuterol use. We selected clinically relevant factors a priori for inclusion in the model without regard for statistical significance (e.g., age, sex, parent-reported previous use of bronchodilator). Other factors were evaluated for possible inclusion in the model if found to be suggestively associated with the outcome in unadjusted analyses (*P*<0.20). The final regression model used logistic regression with clustered standard errors to adjust for potential non-independence of observations within hospitals, analyzing data as a panel by site. We reported results as odds ratios (ORs) with 95% CIs.

## RESULTS

Among 1,016 enrolled infants admitted to hospitals with bronchiolitis, 1,008 (99%) had data regarding pre-admission albuterol use and formed our analytic cohort. In this cohort, the median age was 3.2 months (IQR, 1.6–6.0 months), 603 (60%) were male, 426 (42%) were non-Hispanic White, 803 (80%) had no prior history of breathing problems, and the most commonly detected viruses were RSV (n=814, 81%) and RV (n=212, 21%) (Table 1). Additionally, 445 infants (44%) had previously used a bronchodilator prior to the pre-admission visit (e.g., for past breathing problems or the index problem).

For most infants, the pre-admission visit was in the ED of the enrolling hospital (n=831, 82%). For the other infants, their pre-admission was in another hospital ED prior to transfering to the enrolling hospital (n=119, 12%), at their primary care provider’s office (n=35, 4%), or in other clinics (e.g., an outpatient clinic or urgent care) (n = 23, 2%). In our cohort, 508 infants (50%) were identified as having been administered inhaled albuterol during their pre-admission visit. Across hospitals, the proportion of pre-admission albuterol usage ranged from 23–84% (*P*<0.001; Figure).

Unadjusted associations between patient characteristics and pre-admission albuterol use are shown in Table 1. Several groups of infants were found to have a higher proportion of pre-admission albuterol use, including older infants (≥2 months of age), infants with a history of breathing problems, and infants with previous use of a bronchodilator (i.e., any parent-reported use of a bronchodilator in the infant’s life) (all *P*<0.001). Likewise, infants whose parents reported symptoms of breathing faster than normal (*P*=0.007), wheezing, retractions, or having stopped breathing in the 24 hours prior to the pre-admission visit (all *P*<0.001) also were more likely to have received pre-admission albuterol. Although pre-admission albuterol use was not associated with the most common bronchiolitis viruses (RSV and RV), infants with human metapneumovirus were more likely to have received pre-admission albuterol compared to infants without human metapneumovirus (*P*=0.02; Table 1).

Several of the unadjusted associations with pre-admission albuterol persisted in the multivariable analysis (Table 2). Compared to infants <2 months of age, those 2.0–5.9 months were more likely to have received pre-admission albuterol (OR 2.09, 95% CI [1.45–3.01]); and infants 6.0–11.9 months were the most likely to have received it (OR 2.89, 95% CI [1.99–4.19]). Other significant predictors of pre-admission albuterol use were previous use of a bronchodilator (OR 1.89, 95% CI [1.24–2.90]) and pre-admission chart documentation of wheeze (OR 3.94, 95% CI [2.61–5.93]).Factors inversely associated with pre-admission albuterol use included the following: ≥7 days since the start of index breathing problem prior to pre-admission (OR 0.66, 95% CI [0.44–1.00]); and parent-reported fever (OR 0.75, 95% CI [0.58–0.96]). To create a more parsimonious final model, we excluded detection of human metapneumovirus since it was not associated with pre-admission albuterol in adjusted analyses (*P*=0.85).For completeness, we also examined inpatient data on albuterol use (i.e., albuterol receipt after the primary outcome of pre-admission albuterol use). Among 508 infants who received pre-admisson albuterol, 193 (38%) were also treated with inhaled albuterol during the first 24 hours of admission; among the 500 infants who did not receive pre-admission albuterol, only 77 (15%) went on to be treated with inhaled albuterol during their first 24 hours of inpatient stay (*P*<0.001).

## DISCUSSION

Among infants hospitalized for bronchiolitis in the U.S. from 2011–2014 we found that albuterol was a commonly used pre-admission treatment. Albuterol use varied more than three-fold across hospitals, ranging from 23–84% of infants. We also identified several patient characteristics that were independently associated with an increased likelihood of pre-admission albuterol use: older age, history of bronchodilator use, and pre-admission chart documentation of wheeze. By contrast, other factors were associated with decreased likelihood of pre-admission albuterol use: symptoms present seven days or longer; and parent-reported fever within 24 hours prior to arrival at the hospital.

Our findings suggest that variation in pre-admission albuterol use is strongly influenced by local policy and/or culture. This is consistent with previous literature, which has established local variation across many therapies, including albuterol, in the management of infants hospitalized for bronchiolitis.^9^,^10^,^17^,^18^ Local policies are shaped in part by the AAP national guidelines on bronchiolitis, which include recommendations on albuterol use. In an earlier version of the AAP guidelines, published in 2006 (prior to study enrollment), a trial of α- or β-adrenergics remained an “option” for all patients with bronchiolitis.^15^ Local variation in albuterol use for bronchiolitis persisted, as shown in recent studies and supported by our data, which was collected from 2011–2014.^17^,^19^ The most recent AAP guidelines were published in November 2014 and now state that “[c]linicians should not administer albuterol (or salbutamol) to infants and infants with a diagnosis of bronchiolitis.”^12^ As our data showed, a large majority of infants who receive bronchodilators for bronchiolitis will do so in the pre-admission setting first, so this recommendation especially affects clinicians working in the hospital ED. The evidence behind this recommendation is therefore important context for our observations of chosen therapies for albuterol.

The AAP’s updated recommendation against albuterol use for bronchiolitis is based on “greater evidence” that showed no benefit in bronchodilator use. Specifically referenced was a 2014 Cochrane meta-analysis of 30 randomized controlled trials (RCT) assessing bronchodilators for “bronchiolitis,” based on diverse definitions and clinical populations. This meta-analysis found that bronchodilators used for bronchiolitis were not effective in improving oxygen saturation, nor in reducing the need for hospitalization, and did not shorten length of illness in the hospital or home. Based on these outcomes, the authors concluded that bronchodilators were “not effective in the routine management of bronchiolitis.”^11^ The analysis had been updated from a meta-analysis previously published in 2006, which had concluded that bronchodilators produced a “modest improvement” in clinical scores.^20^ This clinical improvement was not found in the most recent analysis, and may have contributed to the shift in the AAP’s recommendation. Both analyses, however, were limited by significant heterogeneity, and noted that all of the included trials were small and that standardized outcomes were not available across the 30 RCTs.^21^–^26^ Furthermore, while the meta-analysis authors’ conclusion was to consider bronchodilators ineffective in treating bronchiolitis, they did distinguish that this recommendation in practice applied only to “first-time wheezers.”^27^

For evidence against albuterol in infants with recurrent wheeze, the AAP guidelines cite Chavasse and colleagues’ 2002 meta-analysis that concluded no benefit was found in the use of short-acting beta-agonists for recurrent wheeze in infants under two years of age. This analysis was also limited by significant heterogeneity, was not specific to bronchiolitis or a clinical setting, and concluded there was overall “conflicting evidence.”^28^ The evidence cited by the 2014 AAP for eliminating a trial of albuterol in bronchiolitis is still therefore limited to small studies with no standardized outcomes and no clear focus on albuterol or infants less than one year of age.

Notwithstanding this evidence, the AAP guidelines acknowledge that a subgroup of infants may have clinical benefit from the effects of albuterol, but this subgroup was not sufficiently defined at the time of the guidelines’ release. The possibility of an unidentified subgroup of responders has been a common refrain in analyses of β_2_-agonists for bronchiolitis; a meta-analysis in 1997 noted the “possibility that β_2_-agonists are particularly effective therapy for certain subgroups of bronchiolitic patients.”^29^ Early papers showing benefit from albuterol use in bronchiolitis were considered by the AAP guidelines to be a result, in part, of including older infants (greater than one year of age).^29^,^30^ Thus far, though, attempts to define a subgroup of responders to albuterol have focused more on the clinical setting of bronchiolitis treatment (e.g., a comparison of hospitals or inpatient/outpatient settings) rather than the patient characteristics of those who appear to respond.^11^,^28^ The site of treatment has similarly been the focus of papers examining overall variability in the management of bronchiolitis.^9^,^14^,^31^

Our results show that subgroups exist to whom clinicians preferentially give albuterol, enough to cause significant variation in albuterol use even when controlling for hospital-specific variation. The characteristics independently associated with pre-admission albuterol use, including older age, previous bronchodilator use, and presentation with wheeze, resemble those of children whose illness is consistent with recurrent wheeze, a potential precursor of childhood asthma.^32^,^33^ In a recent latent class analysis by our group, a statistical method used on continuous or categorical variables to identify unknown classes, we examined the heterogeneity of 2,500 children (<24 months of age, median age 5.8 months) with bronchiolitis to formally study the issue.^34^ Briefly, we identified a distinct cluster (“Profile A”) of infants who were older (>6 months), had a history of wheeze, and a higher rate of bronchodilator use. Together with our current results (and previous studies), we believe that there is a clinical subgroup of infants with bronchiolitis that has been identified now through two different methods: objective statistical analysis and observed clinician choices of therapy.^35^,^36^ Guidelines that restrict the use of albuterol in all bronchiolitis patients without specifically addressing these patient characteristics are not targeting a significant source of the variation that they aim to reduce. Translational work is needed to further refine these patient characteristics.

The AAP guidelines also base their recommendation against albuterol on the lack of an appropriate objective measure to identify a response of bronchiolitis to β_2_-agonists. We recognize that an objective measure for assessing short-term response to bronchodilators in an infant with bronchiolitis is not available for clinical use; however, such tools are available for research purposes and in non-acute settings.^37^–^40^ In addition, objective clinical scores for this purpose have yet to be widely adopted, and have not been shown to change prescribing practices for bronchodilators in bronchiolitis.^41^ However, lack of an objective measure serves as only more reason to better understand patterns in clinicians’ subjective use of albuterol.^42^ Our inability to measure a benefit does not mean it is insignificant.

We suggest that future trials of albuterol for bronchiolitis could be targeted to patients with characteristics consistently identified by clinicans as potential responders, who as a subgroup may have contributed to the clinical benefit shown in earlier generalized trials of albuterol for bronchiolitis.^29^,^30^ Identifying children as possible responders to albuterol would allow for a reduction in variation of the use of albuterol for bronchiolitis, without losing entirely its potential therapeutic benefit. At the same time, future trials would allow this subgroup to be more precisely defined in order to avoid inappropriately labeling children as requiring more intensive treatment.

## LIMITATIONS

In our analysis, the factors we evaluated for association with pre-admission albuterol use were predominately limited to those collected during a single intake visit, including a parent interview, and could not account for all possible sources of demographic and clinical variation in pre-admission albuterol use. However, our data were supplemented with medical record reviews for further information pertaining to the pre-admission visit and hospitalization. Another limitation is that we did not collect data on the presence of clinical decision support or local quality improvement efforts, and thus,were unable to address how these may have affected hospital-specific rates of albuterol use. However, the multivariable analysis controlled for the clustering of clinician use of albuterol by hospitals, so these efforts would be accounted for in our primary result. Another limitation is that our study did not include patients who presented with bronchiolitis to the ED or another pre-hospital setting but were not later admitted to the hospital. We did not seek to describe the relationship of albuterol with rates of admission. This could be an area for future study. Finally, our study was not designed to address clinical outcomes of albuterol use, as there is no objective clinical measurement for improvement in the pre-admission setting.

## CONCLUSION

This prospective, multicenter, multiyear study of >1,000 infants hospitalized for bronchiolitis showed more than three-fold variation across hospitals in the use of albuterol as a pre-admission treatment from 2011–2014. Several other factors were shown to be associated with albuterol use, including age, presence of wheezing documented in pre-admission chart, and previous use of a bronchodilator. While variation of albuterol use has been reported in previous studies, they have not addressed patient characteristics associated with albuterol use. Given the most recent publication of AAP guidelines recommending against any albuterol use to treat bronchiolitis in infants,^12^ defining a possible subgroup of responders is of renewed importance. Factors that were associated with pre-admission albuterol use – based on clinical data, and supported by recent cluster analyses^34^ – suggest a promising area for future investigation of the targeted use of pre-admission albuterol among a subset of infants with bronchiolitis.

## Figures and Tables

**Figure:**
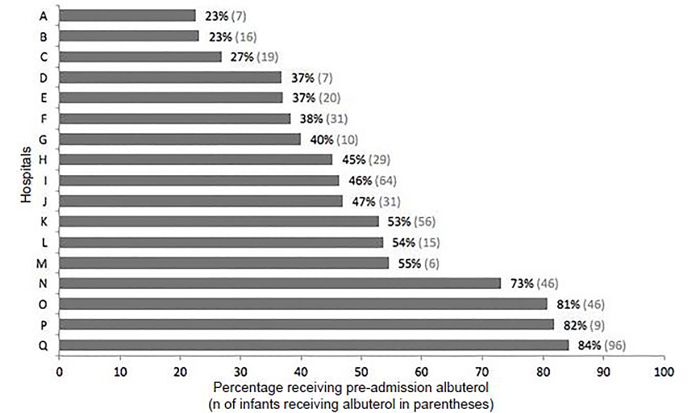
Pre-admission treatment with inhaled albuterol among infants hospitalized for bronchiolitis, by hospital of enrollment.

**Table 1:** Characteristics of infants hospitalized for bronchiolitis and pre-admission albuterol use.

Characteristics	All (n=1008) n (%)	Did not receive pre-admission albuterol (n=500) n (%)	Received pre-admission albuterol (n=508) n (%)	P-value
Age at enrollment in months, median (IQR)	3.2 (1.6–6.0)	2.3 (1.3–4.0)	4.5 (2.5–7.2)	<0.001
Age at enrollment in months				<0.001
<2.0 months	305 (30%)	214 (43%)	91 (18%)	
2.0–5.9 months	451 (45%)	208 (42%)	243 (48%)	
≥6 months	252 (25%)	78 (6%)	174 (34%)	
Sex				0.43
Male	603 (60%)	293 (59%)	310 (61%)	
Female	405 (40%)	207 (41%)	198 (39%)	
Race/ethnicity				0.27
Non-Hispanic white	426 (42%)	226 (45%)	200 (39%)	
Non-Hispanic black	239 (24%)	116 (23%)	123 (24%)	
Hispanic	305 (30%)	140 (28%)	165 (32%)	
Other	38 (4%)	18 (4%)	20 (4%)	
Insurance				0.34
Private	388 (39%)	197 (40%)	191 (38%)	
Public	601 (60%)	290 (58%)	311 (61%)	
None	17 (2%)	11 (2%)	6 (1%)	
Parental history of asthma for either or both parents	343 (34%)	157 (31%)	186 (37%)	0.08
Premature birth (≤37 weeks)	185 (18%)	97 (19%)	88 (17%)	0.39
Number of breathing problems prior to admission				<0.001
0	803 (80%)	420 (84%)	383 (75%)	
1	159 (16%)	71 (14%)	88 (17%)	
2	46 (5%)	9 (2%)	37 (7%)	
Previous use of bronchodilator	445 (44%)	158 (32%)	287 (57%)	<0.001
Number of days since start of current breathing problem prior to pre-admission				0.35
0–6 days	879 (87%)	431 (86%)	448 (88%)	
≥7 days	129 (13%)	69 (14%)	60 (12%)	
Symptoms in 24 hours prior to arrival at hospital, as reported by parents				
Cough	968 (96%)	477 (95%)	491 (97%)	0.31
Runny nose	685 (68%)	333 (67%)	352 (69%)	0.36
Fever	501 (50%)	239 (48%)	262 (52%)	0.23
Hoarseness	552 (55%)	261 (52%)	291 (57%)	0.11
Breathing faster than normal	883 (88%)	424 (85%)	459 (90%)	0.007
Wheezing	728 (72%)	323 (65%)	405 (80%)	<0.001
Retractions	724 (72%)	341 (68%)	383 (75%)	0.01
Stopped breathing	85 (8%)	57 (11%)	28 (6%)	0.001
Pre-admission visit				
Presence of apnea				<0.001
No or not documented	952 (94%)	459 (92%)	493 (97%)	
Yes	56 (6%)	41 (8%)	15 (3%)	
Presence of wheezing				<0.001
No	361 (36%)	262 (52%)	99 (19%)	
Yes	599 (59%)	202 (40%)	397 (78%)	
Not documented	48 (5%)	36 (7%)	12 (2%)	
Initial respiratory rate per minute, median (IQR)	48 (40–60)	48 (40–60)	49 (40–60)	0.23
Initial oxygen saturation by pulse oximetry				0.47
<90%	91 (9%)	40 (8%)	51 (10%)	
90%–93.9%	154 (16%)	75 (15%)	79 (16%)	
≥94%	747 (75%)	378 (77%)	369 (74%)	
Virology				
Number of pathogens detected*				0.003
0	27 (3%)	16 (3%)	11 (2%)	
1	699 (69%)	368 (74%)	331 (65%)	
≥2	282 (28%)	116 (23%)	166 (33%)	
RSV	814 (81%)	409 (82%)	405 (80%)	0.40
RV	212 (21%)	102 (20%)	110 (22%)	0.63
hMPV	56 (6%)	19 (4%)	37 (7%)	0.02

*IQR*, interquartile range; *ED*, emergency department; *bpm*, beats per minute; *ABG*, arterial blood gas; *IV*, intravenous; *CPAP*, continuous positive airway pressure; *ICU*, intensive care unit; *RSV*, respiratory syncytial virus; *RV*, rhinovirus; *hMPV*, human metapneumovirus.

Data are presented as n (%) unless otherwise specified.

* Pathogens tested: RSV types A and B; RV; hMPV, parainfluenza virus types 1, 2, and 3; coronaviruses OC43, 229E, HKU1, and NL63; enterovirus; bocavirus type 1, influenza virus types A and B; adenovirus; *B. pertussis; and M. pneumoniae.*

**Table 2:** Multivariable predictors of pre-admission albuterol use for bronchiolitis.

Characteristics	OR	95%CI		P-value
Age at enrollment in months				
<2.0 months	1.00	reference		
2.0–5.9 months	2.09	1.45	3.01	<0.001
≥6 months	2.89	1.99	4.19	<0.001
Sex				
Male	1.00	reference		
Female	1.06	0.74	1.52	0.76
Race/ethnicity				
Non-Hispanic white	1.00	reference		
Non-Hispanic black	0.94	0.64	1.37	0.74
Hispanic	1.36	0.51	3.65	0.54
Other	1.21	0.53	2.75	0.65
Insurance				
Private	1.00	reference		
Public	1.12	0.75	1.66	0.57
None	0.63	0.20	2.00	0.44
Parental history of asthma for either or both parents	1.12	0.80	1.59	0.51
Premature birth (≤37 weeks)	0.82	0.56	1.21	0.32
Number of breathing problems prior to admission				
0	1.00	reference		
1	0.88	0.54	1.43	0.60
2	1.76	0.85	3.64	0.13
Previous use of bronchodilator	1.89	1.24	2.90	0.003
Number of days since start of current breathing problem prior to pre-admission				
0–6 days	1.00	reference		
≥7 days	0.66	0.44	1.00	0.049
Fever in 24 hours prior to arrival at hospital	0.75	0.58	0.96	0.02
Stopped breathing in 24 hours prior to ED arrival	0.65	0.41	1.03	0.07
Presence of wheezing (pre-admission chart)				
No	1.00	reference		
Yes	3.94	2.61	5.93	<0.001
Not documented	1.03	0.40	2.61	0.96
Number of pathogens detected*				
0	0.79	0.35	1.78	0.57
1	0.87	0.59	1.28	0.48
≥2	1.00	reference		

*OR* denotes odds ratio; *CI*, confidence interval; *ED*, emergency department.

* Pathogens tested: RSV types A and B; RV; hMPV, parainfluenza virus types 1, 2, and 3; coronaviruses OC43, 229E, HKU1, and NL63; enterovirus; bocavirus type 1, influenza virus types A and B; adenovirus; *B. pertussis; and M. pneumoniae.*
